# Biosynthesis of Silver Nanoparticles Mediated by Extracellular Pigment from *Talaromyces purpurogenus* and Their Biomedical Applications

**DOI:** 10.3390/nano9071042

**Published:** 2019-07-21

**Authors:** Sharad Bhatnagar, Toshiro Kobori, Deepak Ganesh, Kazuyoshi Ogawa, Hideki Aoyagi

**Affiliations:** 1 Life Science and Bioengineering, Graduate School of Life and Environmental Sciences, University of Tsukuba, 1-1-1, Tennodai, Tsukuba 305-8572, Ibaraki, Japan; 2 Food Research Institute, National Agriculture and Food Research Organization, 2-1-2, Kannondai, Tsukuba 305-8642, Ibaraki, Japan; 3 Faculty of Life and Environmental Sciences, University of Tsukuba, 1-1-1, Tennodai, Tsukuba 305-8572, Ibaraki, Japan; 4 Microbiology Research Center for Sustainability (MiCS), University of Tsukuba, 1-1-1, Tennodai, Tsukuba 305-8572, Ibaraki, Japan

**Keywords:** *Talaromyces purpurogenus*, silver nanoparticles, anti-cancer activity, anti-microbial activity

## Abstract

In recent years, green syntheses have been researched comprehensively to develop inexpensive and eco-friendly approaches for the generation of nanoparticles. In this context, plant and microbial sources are being examined to discover potential reducing agents. This study aims to utilize an extracellular pigment produced by *Talaromyces purpurogenus* as a prospective reducing agent to synthesize silver nanoparticles (AgNPs). Biosynthesized AgNPs were characterized by transmission electron microscopy (TEM), dynamic light scattering (DLS), electron probe micro analyser (EPMA), and zeta potential. The pigment functional groups involved in the generation of AgNPs were investigated using Fourier transform infrared spectroscopy. TEM images showed that the generated nanoparticles were spherical, hexagonal, rod-shaped, and triangular-shaped with a particle size distribution from 4 to 41 nm and exhibited a surface plasmon resonance at around 410 nm. DLS and zeta potential studies revealed that the particles were polydispersed and stable (−24.8 mV). EPMA confirmed the presence of elemental silver in the samples. Biosynthesized AgNPs exhibited minimum inhibitory concentrations of 32 and 4 μg/mL against *E. coli* and *S. epidermidis*, respectively. Further, cytotoxicity of the AgNPs was investigated against human cervical cancer (HeLa), human liver cancer (HepG2), and human embryonic kidney (HEK-293) cell lines using 5-fluorouracil as a positive control. A significant activity was recorded against HepG2 cell line with a half-maximal inhibitory concentration of 11.1 μg/mL.

## 1. Introduction

Owing to the recent rise in the development of environment-friendly technologies, green nanotechnology has become an area of focus, leading to an exploration of various avenues in the search for natural reducing agents. Plants and microbes have become an essential target in this quest because of their ubiquity. Several plant extracts such as *Aloe vera* [1], *Piper nigrum* [2], *Eucalyptus globulus* [3], *Sida cordofolia* [4], *Rosa damascena* [5], and *Cinnamomum camphora* [6] have been used for silver nanoparticles (AgNPs) generation. Bacteria and fungi have also been used prominently for the reduction of metal salts to nanoparticles. Whole cells and cellular products have been used for synthesizing nanoparticles, such as *Pseudomonas stutzeri* AG259 [7], *Brevibacterium casei* [8], *Fusarium oxysporum* [9], *Aspergillus flavus* [10], and cell-free extracts of *Bacillus subtilis* [11], *Aspergillus fumigatus* [12], and *Cladosporium cladosporioides* [13]. Mukherjee et al. demonstrated a fungal enzymes/protein-mediated generation of nanoparticles [14,15]. Because fungal cells can potentially produce higher amounts of protein than that produced by bacterial cells, the potential for an enhanced yield of nanoparticles becomes higher [16]. Extracellular components such as cell-free broth and extracts are easier to use and can help in minimizing downstream processing.

In this light, extracellular products produced by microbial cells can help in economizing nanoparticle synthesis because the extraction steps can be circumvented. This, in turn, puts focus on the search for extracellular products that are inexpensive and easier to produce and purify. Therefore, pigments derived from microbes have been recently attracting considerable attention for their reduction ability. *Monascus purpureus* [17,18], *Nostoc linckia*, and *Nostoc carneum* [19,20] have been used in the production of pigment for metallic nanoparticle generation. Red and orange monascus pigments were used for the generation of silver and gold nanoparticles in the former studies, whereas the latter utilized phycocyanin and phycoerythrin for silver nanoparticle generation. These studies employed photochemical reduction for nanoparticle synthesis. The nanoparticles produced in these studies were examined for various applications such as antimicrobial activity, biofilm inhibition, heavy metal ion detection, and in-vitro and in-vivo anticancer activity. However, the extraction methods involve the use of organic solvents and/or cell destruction because pigment production is, in general, intracellular. In contrast, *Talaromyces purpurogenus* (*T. purpurogenus*) can produce a high amount of extracellular *Monascus*-like pigments, which negates the need for extensive extraction procedures. *Monascus*-like pigments are a group of azaphilone mixtures containing yellow, orange, and red pigments [21]. Six major monascus pigments include the following: monascin and ankaflavin for yellow pigments; rubropunctatin and monascorubrin for orange pigments; and rubropunctamine and monascorubramine for red pigments. Although these are fundamental pigments produced by *Monascus* species, more than 50 other compounds have been identified as *Monascus* pigments [22]. Several researchers have reported the production of pigments from various *Talaromyces* species, highlighting the fact that this ascomycota has an ability to produce a high amount of water-soluble extracellular pigments and that these pigments comprise of a mixture of various *Monascus*-like pigments [23,24,25,26].

This study attempted to exploit the reduction ability of *T. purpurogenus* extracellular composite pigment for green synthesis of AgNPs. The biosynthesized AgNPs were characterized and their biomedical applications were studied.

## 2. Materials and Methods

### 2.1. Materials

Silver nitrate (AgNO_3_), sucrose, yeast extract, peptone, magnesium sulphate heptahydrate (MgSO_4_·7H_2_O), dipotassium hydrogen phosphate (K_2_HPO_4_), sodium nitrate (NaNO_3_) and Ethanol (99%, special grade) were procured from Fujifilm Wako Pure Chemical Corporation, Chuo-Ku, Osaka, Japan.

### 2.2. Talaromyces Purpurogenus Growth Conditions and Pigment Production

*T. purpurogenus* strain was acquired from Cell Cultivation Lab, Life and Environmental Sciences, University of Tsukuba, Tsukuba, Ibaraki, Japan. Ten millilitres of spore suspension (3 × 10^6^/mL) was used to initiate inoculum production in preculture media. The preculture media consisted of 30 g/L sucrose, 5 g/L yeast extract, 1 g/L K_2_HPO_4_, 0.3 g/L NaNO_3_, 0.05 g/L KCl, 0.05 g/L MgSO_4_·7H_2_O, and 0.001 g/L FeSO_4_·7H_2_O. Flasks were incubated at 30 °C and 150 rpm for 24 h in dark conditions. Following the preparation of preculture media, the production media was prepared with the following composition: 50 g/L sucrose, 25 g/L peptone, 2 g/L K_2_HPO_4_, 2 g/L MgSO_4_·7H_2_O, 1 g/L NaNO_3_, 0.05 g/L KCl, and 0.001 g/L FeSO_4_·7H_2_O. Five percent of the inoculum volume was used to commence pigment production. The flasks were incubated at 30 °C at 150 rpm for 10 days in dark conditions. The pH values of both preculture and production media were adjusted to 5 using 6N hydrochloric acid (HCl).

### 2.3. Extraction of Extracellular Pigment

The culture broth (10 mL) was centrifuged at 6700 *g* for 20 min to separate the extracellular pigment from the biomass. The obtained supernatant was mixed with 70% ethanol in 1:1 ratio for 3 h. The mixture was again centrifuged at 6700 *g* at 4 °C for 20 min to precipitate impurities and other medium components. The supernatant containing the pigment was concentrated using a rotary vapor evaporator, then dissolved in 70% Ethanol and filtered using a 0.45 μm filter (Advantec Toyo Kaisha, Otowa, Tokyo, Japan). The final product was stored at 4 °C until further use.

### 2.4. AgNPs Synthesis Using Pigment

The extracted pigment was used to produce AgNPs. In brief, a 5 mL reaction mixture containing 0.5 g/L pigment and 2 mM of AgNO_3_ was prepared. Before the addition of an AgNO_3_ precursor, the pH of this mixture was adjusted to 12 using a 5 N sodium hydroxide (NaOH) solution. The obtained reaction mixture was vortexed gently for uniform mixing and was incubated at 28 °C with 2000 lux of light for 48 h. Afterwards, nanoparticle generation was verified by visual change of colour and UV-Visible spectroscopy (V-550 spectrophotometer, JASCO, Hachioji, Tokyo, Japan). A silver nitrate solution without the pigment and 0.5 g/L pigment solution without the precursor salt were used as controls. The samples were diluted 30 times before performing the analysis.

### 2.5. Optimization of Nanoparticle Production

This experiment was performed to identify the maximum amount of AgNO_3_ that can be reduced by 0.5 g/L pigment and the time required for reduction. To determine the optimum precursor concentration, a 20 mL reaction mixture containing the pigment and various AgNO_3_ concentrations between 2 mM to 20 mM was prepared in 50 mL Erlenmeyer flasks. The data were expressed in terms of absorption maxima (A_max_), wavelength maxima (λ_max_), and full width at half maxima (FWHM), which represent the spectroscopic yield, size, and size distribution or polydispersity, respectively [27]. A time course study was conducted to estimate the time required for complete reduction of bulk silver. Reaction time is an important factor in the morphology of the nanoparticles being generated. During the crystal growth, the particles may fuse together to give rise to various morphologies. Therefore, the determination of the optimum reaction time becomes essential. For the time course study, a 25 mL reaction mixture containing 0.5 g/L pigment and optimum concentration of AgNO_3_ was prepared in a conical flask and samples were withdrawn at 0, 2, 4, 6, 12, 24, and 36 h. These samples were diluted 60 times before performing UV-Vis spectroscopy analysis to ensure that the maximum value of absorption remains less than two, and the spectra were normalized to determine the FWHM values.

### 2.6. Characterization of Produced Nanoparticles

The produced nanoparticle suspension was centrifuged at 8400 *g* for 10 min, and the resultant pellet was washed twice with Milli-Q water. The final pellet was dissolved in Milli-Q water and kept at 4 °C. A part of the purified nanoparticles was lyophilized to estimate the dry weight. Several techniques were used for the characterization of the nanoparticles. UV-Visible spectroscopy was performed to detect the presence of nanoparticles; transmission electron microscopy (TEM) and dynamic light scattering (DLS) were conducted to estimate the particle size; Fourier transform infrared (FTIR) spectroscopy was carried out for identification of the pigment functional groups involved in the reduction of bulk silver; electron probe microanalysis (EPMA) was performed to detect the existence of silver in the nanoparticles; and zeta potential was investigated to estimate the stability of the colloidal suspension of particles.

#### 2.6.1. TEM

A carbon-coated formvar film was used to prepare nanoparticle samples for TEM analysis. The samples deposited on formvar film were air dried and placed on a copper grid for imaging under a JEOL JEM-2100F transmission electron microscope (Akishima, Tokyo, Japan) at 200 kV.

#### 2.6.2. DLS and Zeta Potential Measurements

A Nano ZS Zetasizer (Malvern Panalytical, Malvern, Worcestershire, UK) was used for measuring the size distribution of the nanoparticle suspensions by DLS and for calculating zeta potential to evaluate the stability of the nanoparticle suspensions. For the analysis of DLS and zeta potential, 3 mL of the nanoparticle suspension was used.

#### 2.6.3. FTIR Spectroscopy

FTIR spectroscopy was performed using a JASCO-FT/IR-6800 spectrophotometer (Hachioji, Tokyo, Japan) over the wavenumber range of 4000–800 cm^−1^. The lyophilized samples were mixed with potassium bromide (KBr) to form pellets for analysis.

#### 2.6.4. EPMA

A JEOL JXA-8530F electron probe micro-analyser (Akishima, Tokyo, Japan) was used for wavelength dispersive spectroscopy (WDS) for composition analysis. WDS measurements are established using Bragg’s law and require the use of multiple crystals as monochromators. The samples were dropped on a coverslip and air dried. Before analysis, the samples were covered with carbon and mounted on a copper grid.

### 2.7. Determination of Minimum Inhibitory Concentration (MIC) and Minimum Bactericidal Concentration (MBC)

MIC and MBC of AgNPs were determined against Gram-positive and Gram-negative microorganisms, namely *Staphylococcus epidermidis* NBRC100911 (*S. epidermidis*) and *Escherichia coli* K 12 (*E. coli*), respectively. The broth microdilution method, performed in a 96-well microplate, was employed with some modifications [28]. Nutrient broth was used to cultivate *S. epidermidis*, whereas LB broth was used for *E. coli*. Both cultivation media were procured from Difco Laboratories Inc., Sparks, MD, USA. AgNPs were tested for concentrations of 0.5–256 μg/mL. Streptomycin was used at the same concentrations as the positive control. Microbial cells were also exposed to the pigment concentration of 1–512 μg/mL to detect any antimicrobial activity. In brief, three to five morphologically-alike colonies grown at 37 °C for 24 h on agar plates were picked up and transferred to sterile fresh broth. This suspension was vortexed briefly to ensure uniform mixing, and the suspension turbidity was adjusted to 0.5 McFarland standard (OD_625_ ≅ 0.08–0.13). The bacterial suspension was diluted 100 times before inoculating with the test/control substance in 1:1 ratio, resulting in a final concentration of 5 × 10^5^ cells/mL. The final volume of the test solution in the 96-well plate was 100 μL, and the last two rows were employed as growth control and sterility control, respectively. Growth control was used to observe the growth of organisms without AgNPs or streptomycin, whereas sterility control was employed to ensure that there was no contamination. The 96-well plates were then incubated at 37 °C for 24 h. The lowest concentration which exhibited no visible growth compared to the control wells was considered the MIC value. To determine the MBC, the suspensions with concentrations higher than the MIC values were plated to check for cell survival, and the least concentration displaying no cell survival was deemed to be MBC. All the experiments were performed in triplicates.

### 2.8. Cell Death Kinetics Study

*S. epidermidis* was used to study the cell death kinetics. Initial inoculum containing 1 × 10^6^ cells/mL was exposed to the MBC dosages of AgNPs and streptomycin in a 96-well plate containing the nutrient broth. The plate was incubated at 37 °C, and the cells were plated at 0, 1, 2, 4, 6, 12, 24, 36, and 48 h in triplicates. The cell count was established, and the data was plotted in terms of the log of colony-forming units per millilitre log_10_(CFU/mL) with respect to time to investigate the cell death kinetics.

### 2.9. Anti-Cancer Activity of Biogenic AgNPs

Cytotoxic activity of biogenic silver nanoparticles was tested against cancerous cell lines HeLa (cervical cancer), HepG2 (Hepatocellular carcinoma) and a non-cancerous cell line HEK-293 (Human embryonic kidney) acting as a control to identify the selectivity of AgNPs. All the cell lines were purchased from JCRB Cell Bank, National Institutes of Biomedical Innovation, Health and Nutrition, Osaka, Japan. 5-Fluorouracil (5-FU), an established anticancer drug, was employed as a positive control [29,30]. The cell lines were also exposed to 500 μg/mL pigment to explore the antiproliferative effect of pigment, if any. Cells were cultured in a collagen-coated 96-well microplate in triplicate to ensure adhesion and proliferation, seeded at a density of 25,000 cells/well, and incubated at 37 °C under 5% CO_2_. HepG2 and HEK-293 cells were cultivated in Dulbecco’s Modified Eagle Medium (DMEM) containing 10% foetal calf serum and 100 μg/mL of penicillin/streptomycin, whereas the HeLa cell line was grown in Ham’s F-12 (10% foetal calf serum) medium. These cells were grown to reach 70% confluency before being exposed to varying concentrations of AgNPs in triplicates from 25 μg/mL to 200 μg/mL. The cell line most affected by the AgNPs was selected for further testing, and the test concentrations were varied from 3.9 μg/mL to 500 μg/mL and 1.5675 μg/mL to 200 μg/mL of 5-FU and AgNPs, respectively. The plates were incubated for 24 h; and then cell counting kit-8 assay (CCK-8, Dojindo Molecular Technology) was performed to measure the cytotoxic activity of AgNPs by determining the absorbance at 450 nm. The assay was performed in accordance with the manufacturer’s protocol. The data obtained were fitted with a four-parameter logistic model using ImageJ software (Version 1.42 q, National Institute of Health, Bethesda, MD, USA).

## 3. Results and Discussion

### 3.1. Silver Nanoparticle Synthesis

**Figure 1:**
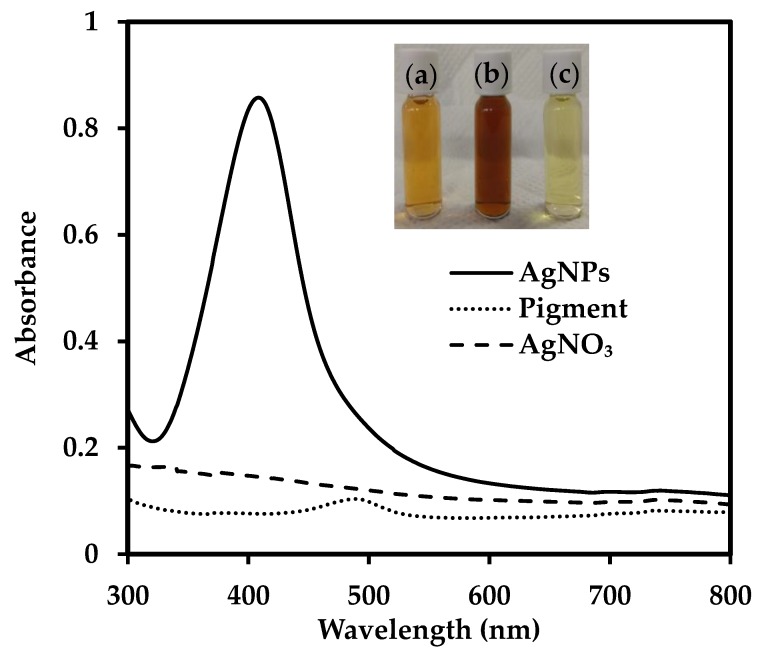
UV-Vis spectrum for AgNPs production using extracellular pigment of *T. purpurogenus* (samples were diluted 30 times for analysis). Inset: (**a**) Pigment, (**b**) Reaction mixture after synthesis, (**c**) Synthesized AgNPs diluted 30 times.

The reduction of silver nitrate to AgNPs using the composite pigment was first observed visually and then spectrophotometrically. The colour of the solution gradually changed from light orange to brown, demonstrating the reduction of silver nitrate (Figure 1a,b). The UV-visible spectrum of the solution exhibited a peak near 410 nm, which is a signature peak for the surface plasmon resonance (SPR) of AgNPs (Figure 1). SPR occurs when conduction electrons on the surface of metallic nanoparticles are excited by a specific wavelength and start oscillating, causing a high absorption or scattering. The diluted synthesized suspension was yellow, which further indicated the presence of AgNPs (Figure 1c). The nanoparticle production by the composite pigment in the presence of light is plausibly due to photolysis wherein light acts as a catalyst, inducing electron transfer and prompting the reduction of metal salt [17,19].

### 3.2. Optimization of Nanoparticle Production

#### 3.2.1. Effect of Precursor Concentration

**Figure 2:**
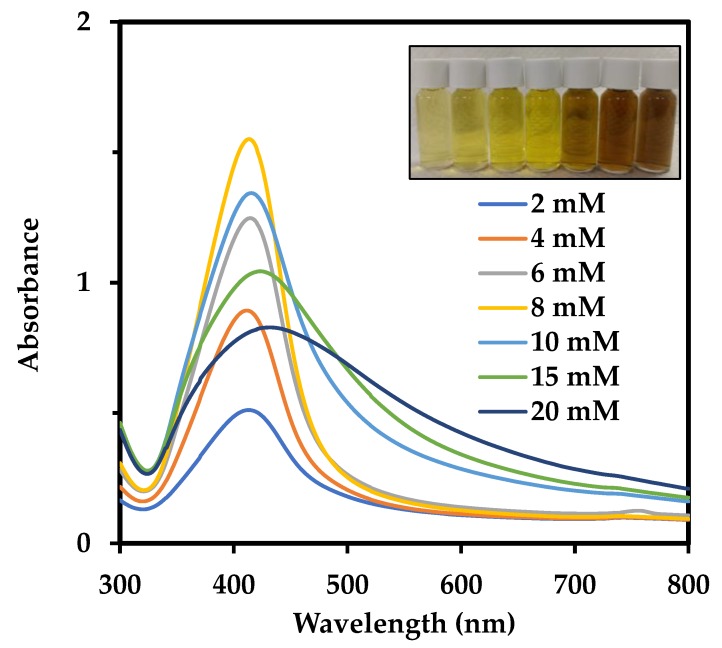
UV-Vis spectra of AgNPs generated from different precursor concentrations (samples were diluted 60 times). Inset shows changes in SPR, indicating an increase in size with increasing concentration. AgNPs diluted 30 times produced from 2, 4, 6, 8, 10, 15, and 20 mM precursor concentration (from left to right).

Precursor concentration plays an important role in the reduction process because it can be one of the limiting factors in nanoparticle synthesis. Since a pigment concentration of 0.5 g/L was consistently used in this study, the amount of precursor that could be reduced to nanoparticles and stabilized by the pigment had to be optimized. The AgNO_3_ concentration was varied from 2 mM to 20 mM. Although change in SPR due to increase in particle size could be discerned in an AgNO_3_ concentration-dependent manner (Inset in Figure 2), UV-visible spectrum of AgNPs prepared with different precursor concentrations showed that the AgNPs production increased steadily till 8 mM and started reducing from 10 mM (Figure 2, Table 1). This suggests that at precursor concentrations below 8 mM, the 0.5 g/L pigment reduced the precursor entirely to AgNPs, but at higher precursor concentrations, AgNO_3_ was not completely reduced by the pigment, leading to the generation of larger sizes of AgNPs due to the aggregation of AgNPs with unreacted AgNO_3_. Moreover, the AgNP suspensions generated from 8 mM onwards were not found to be stable during storage. Since the reduction ability of pigment was intact till 8 mM, the aggregation could have occurred due to a lack of stabilizing agents with respect to the number of particles being produced. Possibly, stabilizers such as surfactants like cetyltrimethylammonium bromide (CTAB) could be introduced at higher precursor concentrations to further improve the stability and yield of the process. A positively-charged surfactant such as CTAB can create a micelle cluster in the vicinity of negatively-charged AgNPs, averting their aggregation [31]. In comparison with other precursor concentrations, the FWHM values increased considerably at 10 mM, 15 mM and 20 mM, indicating an increase in size polydispersity of the product. To reconcile high yield, small particle size, and stability, therefore, 6 mM AgNO_3_ together with 0.5 g/L pigment was chosen to be optimal for AgNPs production. The yield at 6 mM concentration was lesser than that at 8 mM concentration, but the final product generated was more stable and could be stored for a longer period of time without the risk of aggregation.

#### 3.2.2. Time Course Study

**Figure 3:**
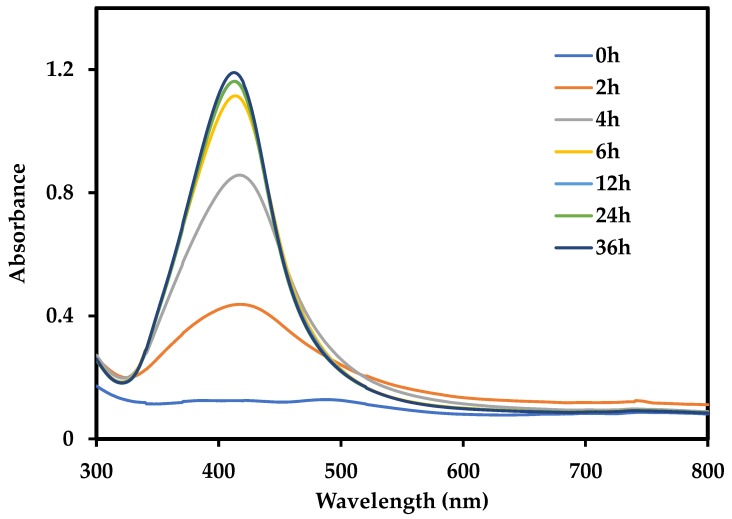
Time course study for generation of AgNPs at 6 mM AgNO_3_ and 0.5 g/L pigment at pH 12. Samples were diluted 60 times.

The time course experiment was performed to ascertain the appropriate reduction time required for the generation of AgNPs. Since 6 mM was determined as the optimal precursor concentration in the earlier experiment, the same was used in this study. The UV-Vis spectrum was recorded after every few hours to check the progress of the synthesis. The increase in absorption of UV-Vis spectrum with time is shown in Figure 3. The synthesis of nanoparticles rapidly progressed for the first 6 h and then the increase was very subtle. After 12 h, the absorbance increased slightly, thereby indicating that the reaction was coming to an end. Therefore, it can be concluded that near-complete reduction was achieved at around 24 h, as judged by the very slight increase in absorbance at later stages around 36 h. No significant changes were observed in the UV-Vis spectrum thereafter. Thus, 24 h was considered the minimum time required to achieve an acceptable degree of reduction for AgNP production. Following the optimization studies, 6 mM AgNO_3_ and 24 h were chosen as the optimum precursor concentration and time, respectively. All further experiments were performed with the nanoparticles obtained using the optimum conditions along with 0.5 g/L pigment.

### 3.3. Characterization of Produced Nanoparticles

#### 3.3.1. Size Estimation by TEM and DLS

**Figure 4:**
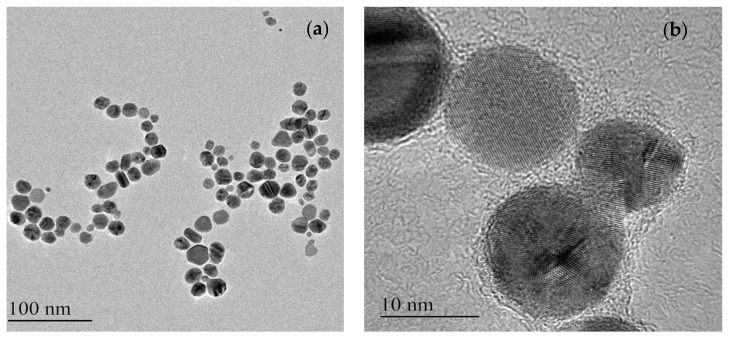
(**a**,**b**) TEM micrographs of the synthesized AgNPs.

TEM analysis illustrated a distribution of spherical, hexagonal, rod-shaped and triangular-shaped particles ranging from 4 to 41 nm (Figure 4a,b).

**Figure 5:**
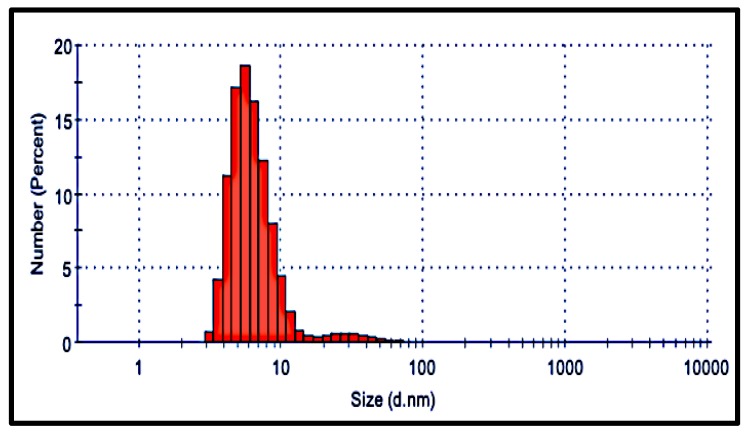
DLS size distribution of AgNPs with number.

Hydrodynamic size estimation of hypothetical spheres by DLS using number distribution with respect to size shows the existence of nanoparticles from 4 to 60 nm. DLS data also indicates the presence of a higher number of particles within 10 nm range and a lesser number of larger particles (Figure 5), which resembles the TEM distribution quite closely. The polydispersity index value (PDI) of suspension was found to be 0.547, which is well below the acceptable range of 0.7. PDI values are dimensionless, ranging from 0 to 1; values lower than 0.05 indicate a monodispersed sample, whereas values higher than 0.7 indicate a broad particle size distribution.

#### 3.3.2. FTIR Analysis

**Figure 6:**
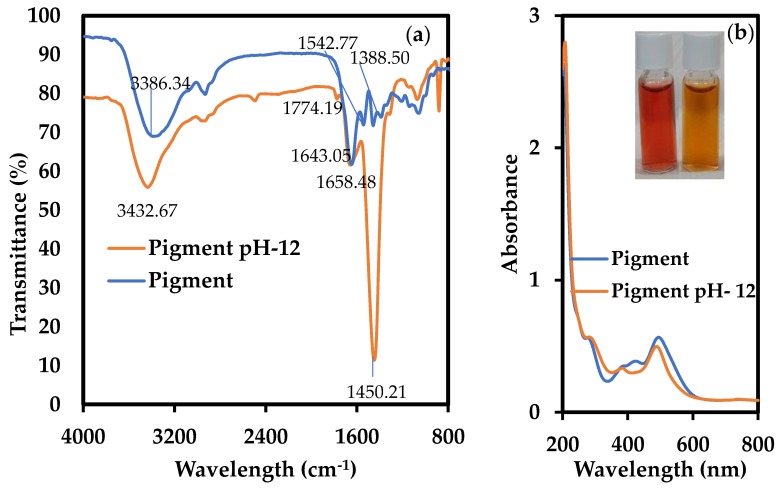
(**a**) FTIR spectrum of pigment without pH change and pigment at pH 12. (**b**) UV-Vis spectrum for both pigment conditions. Inset in (**b**) Change in pigment colour: 0.5 g/L pigment without pH change (left), at pH 12 (right).

FTIR examination was carried out to determine the functional groups present in the pigment, which may have played a vital part in the reduction and capping of AgNPs. Because AgNPs were generated in alkaline conditions, FTIR analysis was performed for the pigment without a pH change, as well as for the pigment at pH 12 to comprehend the change caused by the increased pH (Figure 6a). Table 2 provides information regarding the peaks found in FTIR analysis and their functional groups.

The vibration bands of -OH, amide group, -CH bending, and C-O/primary alcohol groups shifted in the FTIR spectrum of the pigment at pH 12, whereas the =CH_2_ asymmetric stretching band was present in the spectra of both the samples. At alkaline pH, new bands appeared at 1774.19 and 879.38 cm^−1^, and the -CH_2_ bending vibration peak became very strong; however, bands corresponding to amide II, C-O, and primary alcohol groups at 1542.77, 1388.50, and 1211.08 cm^−1^, respectively, disappeared. Therefore, it can be argued that the properties of functional groups of the pigment changed depending on the pH value. The visual change in colour of the pigment at a pH of 12 also evidences the change in optical and absorption properties (Figure 6b, inset). In addition, the UV-Vis spectrum clearly shows shifts in peaks, thereby indicating changes in its functional groups at high pH values. Verma and Mehata (2016) showed that the *Azadiractha indica* extract at pH 13 could reduce the precursor to AgNPs [32]. They concluded that this might be due to an increase in the bioavailability of functional groups in the reaction mixture caused by the change in pH and could be the reason for effective synthesis. In this study, the changes in FTIR spectra and UV-Vis spectra might indicate increased bioavailability, leading to increased reduction ability of functional groups. Nevertheless, the difference in the reduction activity cannot be solely explained by these changes. Previously, NaOH was revealed as an accelerator in the AgNP generation process. Nishimura et al. (2011) showed that NaOH not only plays an important role in the formation of intermediates in Ag^+^ reduction, but also increases the reduction and nucleation rates by oxidizing the reducing agent itself, while using sodium acrylate as a reducing as well as capping agent [33]. Similarly, these factors might account for the better generation of AgNPs by the pigment in alkaline conditions. Further study is essential to realize the mechanisms involved in this process.

The presence of -OH groups along with phenolic/alcoholic groups, and C–O groups with amide groups have been known to induce reduction and stabilization/capping, respectively [34,35]. Phenolic groups take part in redox reactions, which leads to the generation of quinones and donating electrons. These electrons in turn reduce the metal ions to nanoparticles [36]. These groups might also be involved in this reduction process too. Since the synthesis of AgNPs is mediated by bioactive groups, they can possibly be utilized in biomedical applications.

#### 3.3.3. EPMA

**Figure 7:**
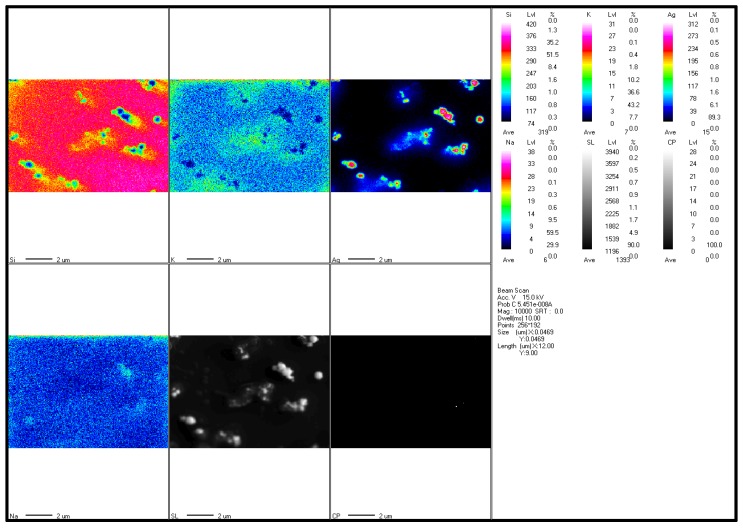
EPMA mapping showing semi-quantitative elemental composition of the targeted area. The scale represents the intensity of the elements present in the mapped area. (Scale bar represents 2000 nm).

The EPMA data qualitatively indicates the existence of elemental silver in the sample (Figure 7). The EPMA maps clearly show the presence of silver in the targeted part of the sample along with other impurities that can be observed in the background. The colour scale represents the relative content of elements present in the targeted sample area. EPMA mapping detected the presence of AgNPs in areas where the sample is present. This is evident in the corresponding scanning micrograph image (SL in Figure 7). The semi-quantitative data and the comparison of the sample with background data for the targeted area are provided as supplementary material (Figure S1 and Table S1).

#### 3.3.4. Zeta Potential

**Figure 8:**
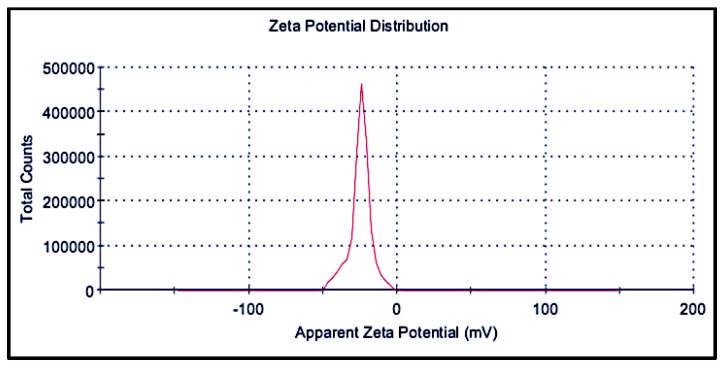
Zeta potential of the colloidal nanoparticle solution after purification.

The zeta potential of the purified nanoparticles was determined to be −24.8 ± 7.2 mV with a conductivity of 0.0119 mS/cm, indicating a moderately stable suspension (Figure 8). The biogenic AgNPs carry an anionic charge on their surface, which could play an important role in their biological activity. Since most cellular membranes are negatively charged, the surface charge may affect the interaction of nanoparticles with the membranes. Positively charged nanoparticles exhibit higher cytotoxicity for the same reason [37]. In a stable colloidal system, the surface effects are more dominant than the bulk effects; therefore, stability depends on factors such as electrostatic interactions, surface charges on the particle, properties of the solvent, and van der Waals forces. The zeta potential of the suspension indicates stability by electrostatic repulsion, as it measures the effective charge present on the nanoparticle surface. The value of zeta potential was estimated by measuring the electrophoretic mobility. For suspensions stabilized only by electrostatic repulsion, suspensions with a zeta potential of ±30 mV are generally treated as highly stable, whereas for suspensions stabilized by steric repulsion, a zeta potential value of ±20 mV is usually accepted as stable [19,38].

### 3.4. MIC and MBC Studies

The anti-microbial activity of the biogenic AgNPs was assessed against a Gram-positive bacterium (*E. coli*) and a Gram-negative bacterium (*S. epidermidis*) by the MIC method. The minimum concentration of AgNPs required to cause inhibition as well as cell death was noted and compared with that of a standard broad-spectrum antibiotic, streptomycin (Table 3). The pigment showed no inhibitory activity at the tested concentrations against either organism. AgNPs seemed to exhibit superior activity against Gram-positive bacteria than against Gram-negative bacteria, which is contrary to the theory that owing to the different cell wall characteristics, AgNPs are less effective towards Gram-positive bacteria than towards Gram-negative bacteria [39]. Gram-negative cells are more vulnerable to AgNPs due to the presence of lipopolysaccharides on their surface that carry a negative charge; this results in higher interaction with the positive silver ions, that eventually results in the degradation of the cell wall. In this case, the stronger action against a Gram-positive bacterium might be due to the presence of bioactive functional groups on the surface of AgNPs. It might also be a case of strain specificity, that is, this particular strain of *S. epidermidis* is more susceptible to the antibacterial action of bio-coated AgNPs.

**Figure 9:**
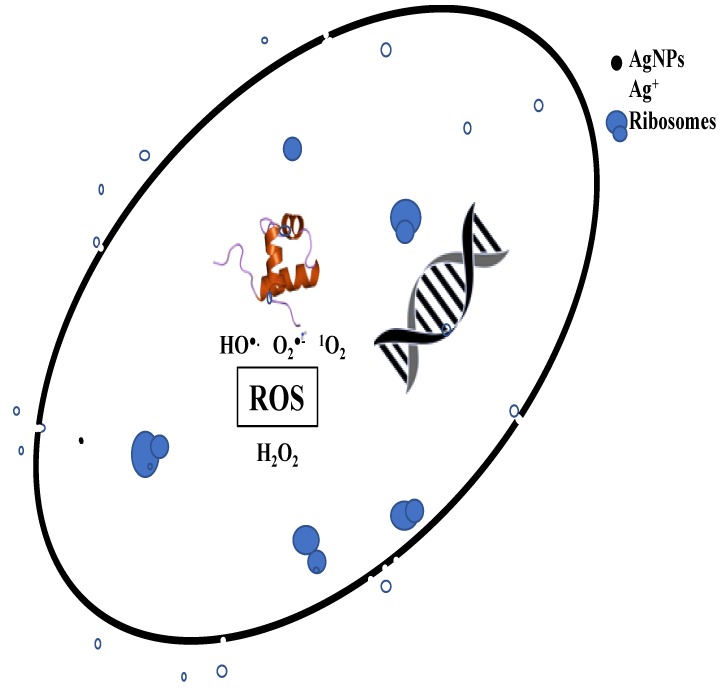
Putative concerted mechanistic model of antibacterial action of AgNPs showing the interaction of AgNPs and silver ions (Ag^+^) with cell membrane, ribosomes, proteins, and DNA leading to ROS production and consequent cell death.

Several concerted mechanisms are responsible for the cytotoxic action of AgNPs against bacterial cells. These mechanisms include the disintegration of cell walls by physical interaction with AgNPs and Ag^+^ ions, followed by the leakage of intracellular components; denaturation of proteins and enzymes by the ions released from AgNPs, leading to the cessation of adenosine triphosphate (ATP) production; and the interaction with DNA and consequent production of reactive oxygen species (ROS), which come in contact with the ribosomes and decrease their activity (Figure 9) [40,41,42,43]. AgNPs might also be able to impede cell respiration by reacting with oxygen and sulfhydryl groups present atop the cell surface, leading to ATP depletion and cell death [44]. In contrast, streptomycin inhibits bacterial cell proliferation by attaching to the 16S rRNA of the 30S subunit, thereby leading to the inhibition of protein synthesis. Other researches have shown that the nanoparticle size is an important factor in terms of penetration power and interaction with the cell membrane [45]. With increasing cases of antibiotic resistance, AgNPs might be able to provide a potential alternative to conventional anti-microbial agents.

### 3.5. Microbial Cell-Death Kinetics Study

**Figure 10:**
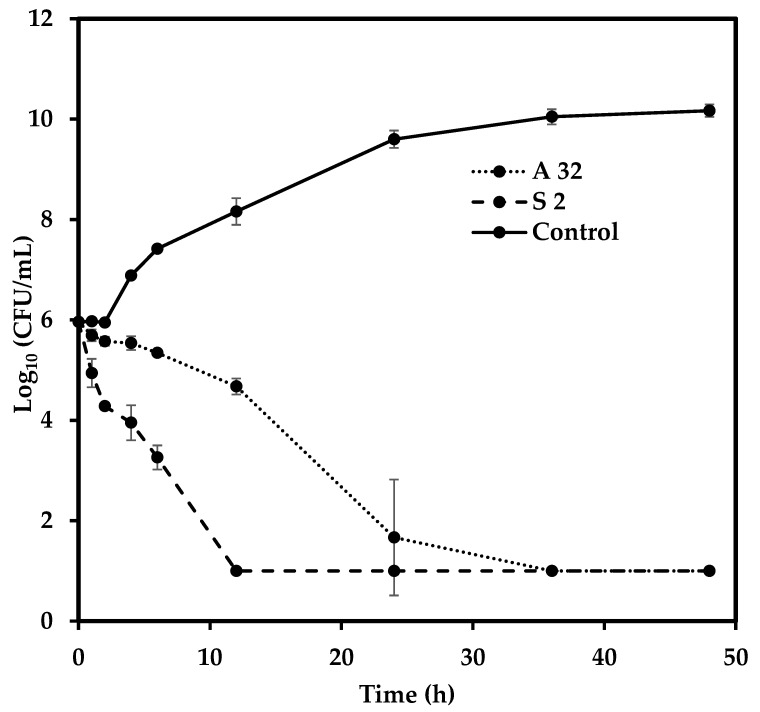
Cell death kinetics for *S. epidermidis.* Error bars represent standard deviation (n = 3). A 32 refers to the MBC dosage for AgNPs (32 μg/mL) and S2 refers to the MBC dosage of streptomycin (2 μg/mL). Control refers to cells grown without AgNPs/Streptomycin.

The microbial cell death kinetics study was conducted at the MBC, the concentration at which cell death occurs. The concentrations of AgNPs and streptomycin used were 32 μg/mL and 2 μg/mL, respectively. The initial cell concentration was 1 × 10^6^ cells/mL. Streptomycin exhibited a faster mode of action compared to AgNPs, reaching zero CFU count in 12 h, while AgNPs required approximately 24 h to reach zero CFU count (Figure 10). In contrast, the control cells achieved a robust growth, entering the log phase after 6 h and the stationary phase after 24 h. The decrease in CFU count for streptomycin was evident only after 2 h, whereas the initial decrease in the case of AgNPs was clearly evident after 6 h. Bactericidal action can be represented in terms of logarithmic reduction, which correlates the CFU of the untreated cells with that of the treated cells. A reduction of greater than 3 log_10_ compared with the initial inoculum can be treated as evidence of bactericidal action [46]. In the case of streptomycin, this reduction was achieved between 6 and 12 h, whereas the AgNPs reached this reduction between 12 and 24 h. The final cell count in both the cases was found to be zero in 1:10 dilution. The data was treated accordingly, assuming the presence of 10 viable cells in the sample, giving the final log_10_(CFU) value as 1. Although AgNPs exhibited slower bactericidal kinetics compared with streptomycin, they both eventually resulted in cell death. As such, in future, it might be prudent to use AgNPs in conjunction with standard antibiotics, so that the efficiency of traditional antibiotics can be merged with the penetration ability of AgNPs along with their own antibacterial ability.

### 3.6. Anti-Cancer Activity of Biogenic AgNPs

**Figure 11:**
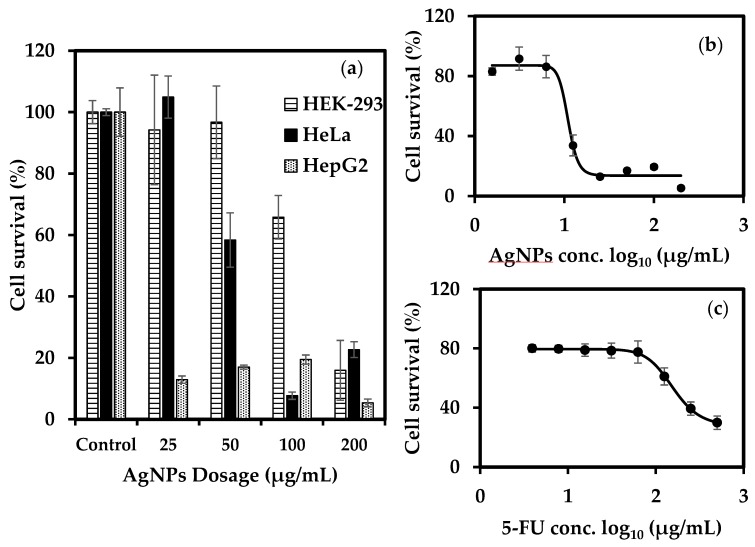
(**a**) Anticancer activity of biofunctionalized AgNPs against various cell lines. (**b**) Effect of AgNPs on HepG2 cell line. (**c**) Effect of 5-FU on HepG2 cell line. Error bars represent standard deviation (n = 3).

Recently, researchers have shown that due to their unique properties, AgNPs possess a therapeutic potential that can be utilized in cancer treatment. Figure 11a shows that, with an increase in nanoparticle concentration, the cell survival decreased for all the cell lines, indicating the cytotoxic effect of AgNPs on cell population. In addition, the data shows that compared to HeLa and HepG2, HEK-293 was more resistant to these effects than HeLa and HepG2, since more than 60% of HEK-293 survived exposure to AgNPs concentration of 100 μg/mL. This indicates that AgNPs are somewhat selective towards cancer cell lines. The nanoparticles affected HepG2 most strongly. Therefore, the HepG2 cell line was selected for studying the anti-cancer activity of AgNPs further in detail. The pigment at 500 μg/mL exhibited no antiproliferative activity against the HEK-293 and HeLa cell line (Figure S2 (I,III)), whereas a weak activity was detected against the HepG2 cell line, where cell survival was almost 80% after exposure to pigment (Figure S2 (II)). No discernible change was observed in the cell morphology after exposure to pigment in any cell line. The presence of bioactive ligands responsible for pigment’s weak cytotoxic activity on the surface of nanoparticles could explain the strong activity of biogenic AgNPs against the HepG2 cell line.

**Figure 12:**
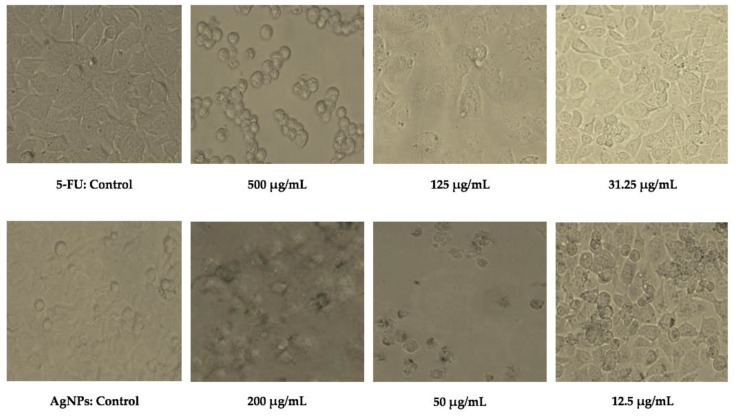
Changes in HepG2 cell morphology after exposure to various concentrations of 5-FU (**top**) and AgNPs (**bottom**).

A four-parameter logistic model curve was plotted using the data obtained after exposing the HepG2 cell lines to various dosages of 5-FU and AgNPs. This curve is useful for biological models such as dose-response and receptor–ligand binding assays. The curve was used to calculate the values of the half-maximal inhibitory concentration (IC_50_), which refers to the concentration of the drug required for 50% cell death. The IC_50_ value of AgNPs (~11.1 μg/mL) was found to be lower than that of 5-FU (154.88 μg/mL), thereby indicating that AgNPs demonstrated a much stronger action against HepG2 cells compared to 5-FU (Figure 11b,c). Figure 12 shows the changes in cell morphology. At high concentrations of 5-FU, almost all the cells are dead, spherical and floating, as compared to the control where the cells are attached to the surface. In the case of AgNPs, the dead cells appear damaged by AgNPs as they exhibit an irregular shape and are clumped together. This might be due to the difference in the mode of action of the two samples. 5-FU mainly acts as a thymidylate synthase inhibitor that interrupts the DNA replication. In contrast, AgNPs seem to have interacted with the cell membrane, causing disruption apart from its other modes of action. As the dosage of both 5-FU and AgNPs is decreased, the cells exhibit a comparably healthier growth which is evident from cell morphology at concentrations of 31.25 μg/mL and 12.5 μg/mL for 5-FU and AgNPs, respectively. At a mid-level dosage of 125 μg/mL for 5-FU, although the cell growth seems less dense compared to the control, the cells are still alive; however, for 50 μg/mL of AgNPs dosage, the cells are dead. This can be corroborated with the cell survival values shown in the curve. The images clearly depict the effect of 5-FU and AgNPs on the HepG2 cell line in a dose-dependent manner. Coupled with the selectivity shown against HEK-293, the results suggest that AgNPs might have a window of opportunity to selectively act against cancer cell lines.

Previously, the researchers have speculated that the primary manner of action of AgNPs could be the interaction of released ions with the cellular components, leading to the production of ROS and oxidative stress that causes apoptotic cell death [47]. In addition, this interaction reduces ATP production and disrupts the functioning of cellular proteins and enzymes such as protein kinase, thereby interfering with cell repair [48]. Apart from this, AgNPs have been shown to constrain angiogenesis, which is an essential step in tumour growth and metastasis [49]. These diverse mechanisms might act in concert for the AgNPs to exert their anti-cancer potential.

The activity of biogenic AgNPs against several cancerous cell lines has been previously reported. El-Naggar et al. [19] prepared phycocyanin-reduced AgNPs that had an IC_50_ value of 27.79 ± 2.3 µg/mL against mammary gland breast cancer (MCF-7), 31.78 ± 2.2 µg/mL against human lung fibroblast (WI38) cell line, and 32.97 ± 1.7 µg/mL against human amnion (WISH) cell lines. Similarly, Anbazhagan et al. [50] synthesized AgNPs using *Cunninghamella echinulata* and tested their cytotoxicity against Vero cell lines, which showed an IC_50_ value of 62.8 µg/mL for AgNPs. This activity was contrasted against the cytotoxic activity of AgNO_3_, which indicated that myco-synthesized nanoparticles were less cytotoxic to normal cells than bulk salts. AgNPs synthesized using *Klebsiella oxytoca* DSM 29614 were coated with exopolysaccharides under the presence and absence of oxygen. The AgNPs synthesized under aerobic conditions exhibited very strong activity against human breast cancer cell lines, SKBR3 and 8701-BC, with IC_50_ values of 5 µg/mL and 8 µg/mL, respectively [51]. In addition, they exhibited IC_50_ values of 20 ± 2, 26 ± 2, and 34 ± 4 µg/mL against human colon cancer cell lines, HT-29, HCT 116, and Caco-2, respectively [51].

Ahmadian et al. [52] showed that chemically produced AgNPs have an IC_50_ value of 75 µg/mL against the HepG2 cell line. In contrast, Justin Packia Jacob et al. [53] estimated an IC_50_ of 31.25 µg/mL against HepG2 cells for AgNPs prepared by reduction using *Piper longum* leaf extracts and hypothesized that this action might be attributed to piperidine present in the leaf extract coating the AgNP surface. These results indicate that the properties of AgNPs generated from reduction using biological compounds might be different from that of the chemically synthesized AgNPs. Moreover, the difference in the nature of bio-reductant imparts different properties to the synthesized nanoparticles. In this study, AgNPs exhibited an IC_50_ value of 11.1 μg/mL, signifying a very strong activity against HepG2 cell lines. This activity is quite comparable to the other values mentioned in the literature. The functional groups and molecules present on the AgNPs surface in conjunction with the penetrative power of nanoparticles, might be responsible for the potent anti-cancer activity of AgNPs produced in this study. Collectively, the data suggests that biogenic AgNPs derived in this study can potentially inhibit cancer cell growth. Although the initial results seem promising, further studies on the mode of action and selectivity of AgNPs are required before AgNPs can be proposed as a suitable addition in the armoury against cancer cells.

## 4. Conclusions

The extracellular pigment of *T. purpurogenus* was successfully utilized as a reducing agent to bio-synthesize AgNPs. A low pigment concentration of 0.5 g/L was required, which provides scope for scalability of the synthesis process. The optimum precursor concentration and reduction time were found to be 6 mM and 24 h, respectively. Characterization of the nanoparticles revealed their size to be in range of 4 to 41 nm. Further exploration is needed to understand the process of AgNP synthesis. The FTIR spectra of the pigment in natural and alkaline conditions were compared to understand the groups involved in the reduction mechanism, and the anti-microbial and anti-cancer activities of the biofunctionalized AgNPs were evaluated. The anti-microbial activity was detected to be more significant against Gram-positive *S. epidermidis*, which was ascribed to the presence of functional groups coating the exterior surface of the nanoparticles. Further research can be conducted for various combinations of antibiotics and AgNPs to understand their synergistic potential in the face of rising antibiotic resistance. The strong activity of the biofunctionalized AgNPs against HepG2 cancer cell line was also attributed to the molecules coating the nanoparticle surface. The mode of action of biogenic AgNPs and the reasons behind selectivity are still unclear and can be studied in detail in the future. More research is required to understand the molecules and functional groups present in the pigment, and to unearth other probable applications of the pigment-mediated biosynthesized AgNPs.

## Figures and Tables

**Table 1:** Characteristics of AgNPs synthesized at various precursor concentrations. Samples were diluted 60 times.

AgNO_3_ Concentration (mM)	A_max_	λ_max_	FWHM
2	0.51	413	90
4	0.89	411	82
6	1.25	414	84
8	1.55	414	81
10	1.34	415	105
15	1.04	423	154
20	0.83	431	199

**Table 2:** Peaks obtained by FTIR analysis and corresponding functional groups.

Pigment		Pigment—pH 12	
Peak (cm^−1^)	Functional Group	Peak (cm^−1^)	Functional Group
3386.39	Broad H-bonded -OH stretching	3432.67	broad -OH bending
2931.27	=CH_2_ asymmetric stretching	2931.27	=CH_2_ asymmetric stretching
1643.05	amide I/-NH bending	1774.19	-C=O group
1542.77	amide II	1658.48	amide group
1457.92	-CH bending	1450.21	strong methylene -CH bending
1388.50	phenol/-OH bending	1072.23	C-O stretch/primary alcohol stretching
1211.08	phenol/C-O- stretching	879.38	-CH vibrations
1056.80	primary alcohol/C-O- stretching		

**Table 3:** MIC and MBC values for AgNPs and Streptomycin.

Organism	Sample	MIC (µg/mL)	MBC (µg/mL)
*E. coli*	AgNPs	32	64
	Streptomycin	8	32
*S. epidermidis*	AgNPs	4	32
	Streptomycin	<0.5	2

## Supplementary Materials

The following are available online at https://www.mdpi.com/2079-4991/9/7/1042/s1, Figure S1: (a) Qualitative EPMA analysis of sample showing presence of Ag between 3 and 4 keV. (b) Comparison with coverslip background showing the presence of impurities, Table S1: Semi-quantitative analysis of the targeted sample area by EPMA, Figure S2: (I) Effect of pigment on HEK-293, (II) HepG2 and, (III) HeLa cell lines. (a) Cell survival after exposure to 500 µg/mL of pigment (n = 3), (b) Cell morphology without exposure to pigment, (c) Cell morphology after exposure to pigment.
