# Supplementary material for: Biosynthesis of Silver Nanoparticles Mediated by Extracellular Pigment from Talaromyces purpurogenus and Their Biomedical Applications
Source: Nanomaterials (Basel). 2019 Jul 21;9(7):1042. doi: 10.3390/nano9071042 (PMC6669664; doi:10.3390/nano9071042)
Supplement: Supplementary file 1 [file nanomaterials-09-01042-s001.pdf]

Supplementary figures

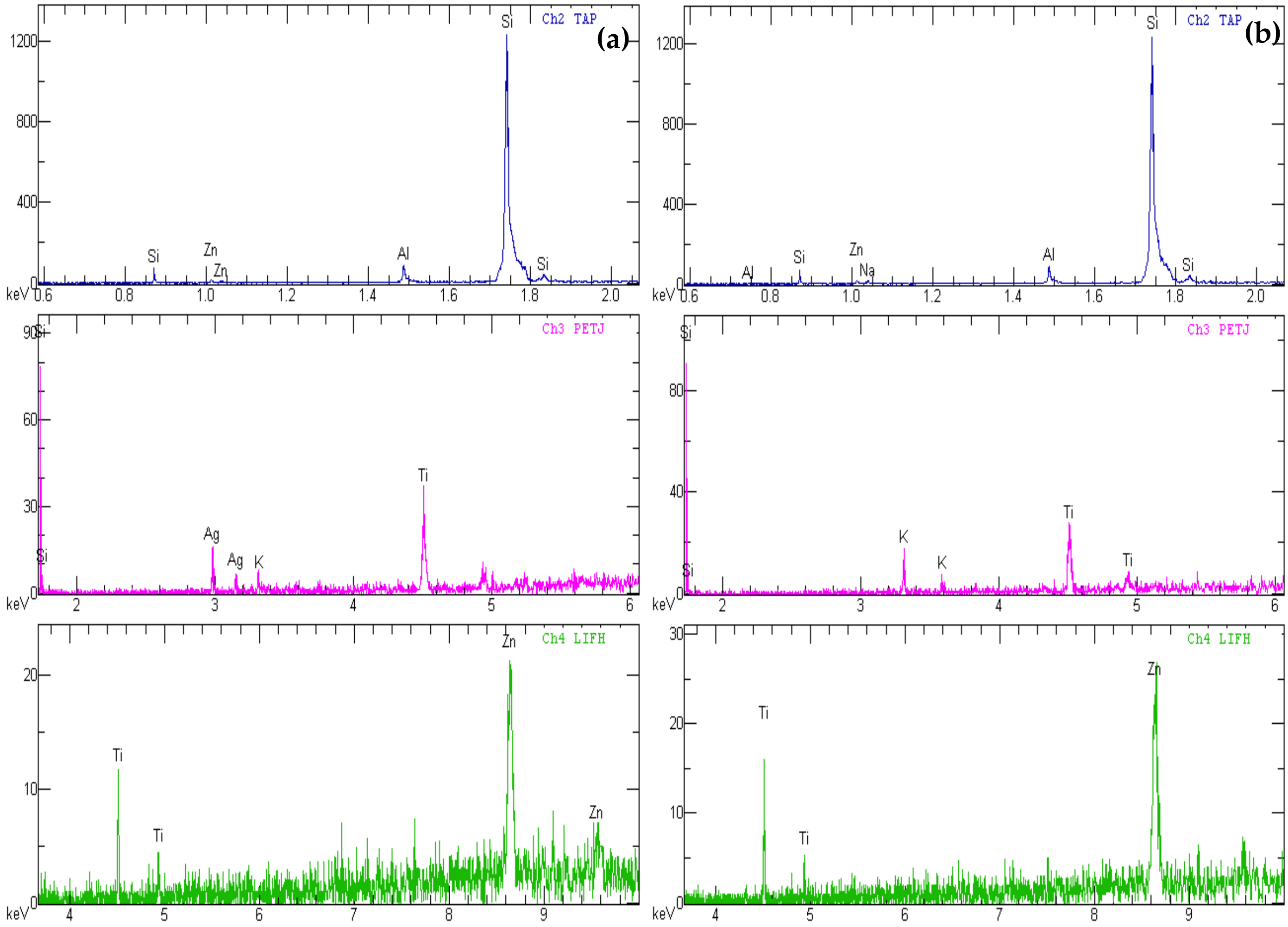

Figure. S1: (a) Qualitative EPMA analysis of sample showing presence of Ag between 3 and 4 keV. (b) Comparison with coverslip background showing the presence of impurities.

Table S1: Semi-quantitative analysis of the targeted sample area by EPMA.

Element	Mass (%)	Atom (%)	K (%)	ZAF
O	47.164	63.7637	19.792	2.227
Na	3.725	3.5044	1.784	1.9512
Al	2.747	2.2019	1.862	1.3784
Si	32.474	25.0076	24.43	1.2422
K	3.582	1.9812	2.917	1.1474
Ti	3.277	1.4795	2.588	1.1829
Zn	4.998	1.654	3.843	1.2154
Ag	2.033	0.4077	1.475	1.2878

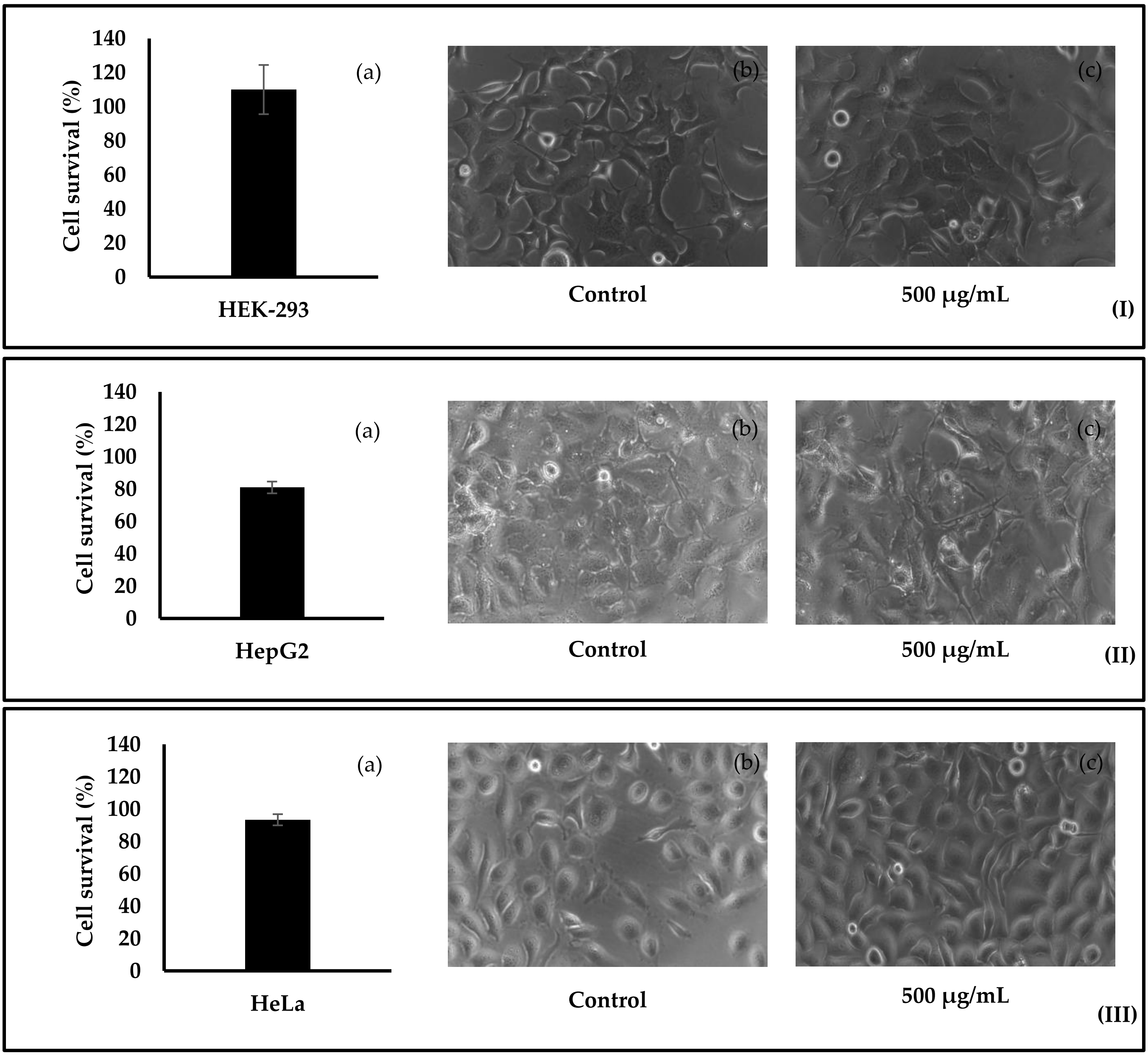

Figure S2: (I) Effect of pigment on HEK-293, (II) HepG2 and, (III) HeLa cell lines. (a) Cell survival after exposure to 500 µg/mL of pigment (n=3), (b) Cell morphology without exposure to pigment, (c) Cell morphology after exposure to pigment.
